# A new localization set for generalized eigenvalues

**DOI:** 10.1186/s13660-017-1388-x

**Published:** 2017-05-15

**Authors:** Jing Gao, Chaoqian Li

**Affiliations:** 1 Department of Mathematics, Guangzhou Vocational College of Technology & Business, Guangzhou, Guangdong 510000 China; 2 School of Mathematics and Statistics, Yunnan University, Kunming, Yunnan 650091 China

**Keywords:** 15A45, 15A48, generalized eigenvalue, inclusion set, matrix pencil

## Abstract

A new localization set for generalized eigenvalues is obtained. It is shown that the new set is tighter than that in (Numer. Linear Algebra Appl. 16:883-898, 2009). Numerical examples are given to verify the corresponding results.

## Introduction

Let $\mathbb{C}^{n\times n}$ denote the set of all complex matrices of order *n*. For the matrices $A, B \in\mathbb{C}^{n\times n}$, we call the family of matrices $A-zB$ a matrix pencil, which is parameterized by the complex number *z*. Next, we regard a matrix pencil $A-zB$ as a matrix pair $(A, B)$ [1]. A matrix pair $(A, B)$ is called regular if $\operatorname{det}(A-zB) \neq0 $, and otherwise singular. A complex number *λ* is called a generalized eigenvalue of $(A, B)$, if

$$\operatorname{det}(A-\lambda B)=0. $$

Furthermore, we call a nonzero vector $x\in\mathbb{C}^{n}$ a generalized eigenvector of $(A, B)$ associated with *λ* if

$$Ax=\lambda Bx. $$

Let $\sigma(A,B)=\{\lambda\in\mathbb{C}: \operatorname{det}(A-\lambda B)=0\}$ denote the generalized spectrum of $(A, B)$. Clearly, if *B* is an identity matrix, then $\sigma(A,B)$ reduces to the spectrum of *A*, *i.e.* $\sigma(A,B)=\sigma(A)$. When *B* is nonsingular, $\sigma(A,B)$ is equivalent to the spectrum of $B^{-1}A$, that is,

$$\sigma(A,B)=\sigma\bigl(B^{-1}A\bigr). $$

So, in this case, $(A, B)$ has *n* generalized eigenvalues. Moreover, if *B* is singular, then the degree of the characteristic polynomial $\operatorname{det}(A-\lambda B)$ is $d < n$, so the number of generalized eigenvalues of the matrix pair $(A, B)$ is *d*, and, by convention, the remaining $n-d$ eigenvalues are ∞ [1, 2].

We now list some notation which will be used in the following. Let $N=\{1,2,\ldots,n\}$. Given two matrices $A=(a_{ij})$, $B=(b_{ij})\in\mathbb{C}^{n\times n}$, we denote

$$\begin{gathered} r_{i}(A)=\sum_{k\in N,\atop k\neq i} \vert a_{ik} \vert ,\qquad r_{i}^{j}(A)=\sum _{k\in N,\atop k\neq i,j} \vert a_{ik} \vert , \\ R_{i}(A,B,z)=\sum_{k\in N,\atop k\neq i} \vert a_{ik}-zb_{ik} \vert ,\qquad R_{i}^{j}(A,B,z)= \sum_{k\in N,\atop k\neq i,j} \vert a_{ik}-zb_{ik} \vert , \\ \Gamma_{i}(A,B)= \bigl\{ z\in \mathbb{C}: \vert a_{ii}-zb_{ii} \vert \leq R_{i}(A,B,z) \bigr\} , \end{gathered} $$

and

$$\begin{aligned} \Phi_{ij}(A,B) =& \bigl\{ z\in \mathbb{C}: \bigl\vert (a_{ii}-zb_{ii}) (a_{jj}-zb_{jj})-(a_{ij}-zb_{ij}) (a_{ji}-zb_{ji}) \bigr\vert \\ & {}\leq \vert a_{jj}-zb_{jj} \vert R_{i}^{j}(A,B,z)+ \vert a_{ij}-zb_{ij} \vert R_{j}^{i}(A,B,z) \bigr\} . \end{aligned}$$

The generalized eigenvalue problem arises in many scientific applications; see [3–5]. Many researchers are interested in the localization of all generalized eigenvalues of a matrix pair; see [1, 2, 6, 7]. In [1], Kostić *et al.* provided the following Geršgorin-type theorem of the generalized eigenvalue problem.

### Theorem 1

[1], Theorem 7

*Let* $A,B\in\mathbb{C}^{n\times n}$, $n\geq2$ *and* $(A,B)$ *be a regular matrix pair*. *Then*

$$\sigma(A,B)\subseteq\Gamma(A,B)=\bigcup_{i\in N} \Gamma_{i}(A,B). $$

Here, $\Gamma(A,B)$ is called the generalized Geršgorin set of a matrix pair $(A, B)$ and $\Gamma_{i}(A,B)$ the *i*th generalized Geršgorin set. As showed in [1], $\Gamma(A,B)$ is a compact set in the complex plane if and only if *B* is strictly diagonally dominant (*SDD*) [8]. When *B* is not *SDD*, $\Gamma(A,B)$ may be an unbounded set or the entire complex plane (see Theorem 2).

### Theorem 2

[1], Theorem 8

*Let* $A=(a_{ij})$, $B=(b_{ij})\in\mathbb{C}^{n\times n}$, $n\geq2$. *Then the following statements hold*:

(i) *Let* $i\in N$ *be such that*, *for at least one* $j\in N$, $b_{ij} \neq0$. *Then* $\Gamma_{i} (A, B)$ *is an unbounded set in the complex plane if and only if* $\vert b_{ii} \vert \leq r_{i}(B)$.
(ii) $\Gamma(A,B)$ *is a compact set in the complex plane if and only if* *B* *is SDD*, *that is*, $\vert b_{ii} \vert > r_{i}(B)$.
(iii) *If there is an index* $i\in N$ *such that both* $b_{ii} =0$ *and*
  $$\vert a_{ii} \vert \leq\sum_{k\in\beta(i), \atop k\neq i} \vert a_{ik} \vert , $$
  *where* $\beta(i)=\{j\in N: b_{ij}=0\}$, *then* $\Gamma_{i}(A, B)$, *and consequently* $\Gamma(A, B)$, *is the entire complex plane*.

Recently, in [2], Nakatsukasa presented a different Geršgorin-type theorem to estimate all generalized eigenvalues of a matrix pair $(A,B)$ for the case that the *i*th row of either *A* (or *B*) is *SDD* for any $i\in N$. Although the set obtained by Nakatsukasa is simpler to compute than that in Theorem 1, the set is not tighter than that in Theorem 1 in general.

In this paper, we research the generalized eigenvalue localization for a regular matrix pair $(A,B)$ without the restrictive assumption that the *i*th row of either *A* (or *B*) is *SDD* for any $i\in N$. By considering $Ax=\lambda Bx$ and using the triangle inequality, we give a new inclusion set for generalized eigenvalues, and then prove that this set is tighter than that in Theorem 1 (Theorem 7 of [1]). Numerical examples are given to verify the corresponding results.

## Main results

In this section, a set is provided to locate all the generalized eigenvalue of a matrix pair. Next we compare the set obtained with the generalized Geršgorin set in Theorem 1.

### A new generalized eigenvalue localization set

#### Theorem 3

*Let* $A=(a_{ij})$, $B=(b_{ij})\in\mathbb{C}^{n\times n}$, *with* $n\geq2$ *and* $(A,B)$ *be a regular matrix pair*. *Then*

$$\sigma(A,B)\subseteq\Phi(A,B)=\bigcup_{i,j=1,\atop i\neq j}^{n} \Bigl\{ \Phi_{ij}(A,B)\cap\Phi_{ji}(A,B) \Bigr\} . $$

#### Proof

For any $\lambda\in\sigma(A,B)$, let $0\neq x=(x_{1},x_{2},\ldots,x_{n})^{T}\in \mathbb{C}^{n}$ be an associated generalized eigenvector, *i.e.*,

1 $$ Ax=\lambda Bx. $$

Without loss of generality, let

$$\vert x_{p} \vert \geq \vert x_{q} \vert \geq\max \bigl\{ \vert x_{i} \vert :i\in N, i\neq p,q\bigr\} . $$

Then $x_{p}\neq0$.

(i) If $x_{q}\neq0$, then from Equality (1), we have

$$a_{pp}x_{p}+ a_{pq}x_{q}+\sum _{k\in N, \atop k\neq p,q } a_{pk}x_{k}=\lambda b_{pp}x_{p}+ \lambda b_{pq}x_{q}+ \lambda \sum_{k\in N, \atop k\neq p,q } b_{pk}x_{k} $$

and

$$a_{qq}x_{q}+ a_{qp}x_{p}+\sum _{k\in N, \atop k\neq q,p } a_{qk}x_{k}=\lambda b_{qq}x_{q}+ \lambda b_{qp}x_{p}+ \lambda \sum_{k\in N, \atop k\neq q,p } b_{qk}x_{k}, $$

equivalently,

2 $$ (a_{pp}-\lambda b_{pp})x_{p}+ (a_{pq}-\lambda b_{pq})x_{q} = - \sum _{k\in N, \atop k\neq p,q} (a_{pk}-\lambda b_{pk})x_{k} $$

and

3 $$ (a_{qq}-\lambda b_{qq})x_{q}+ (a_{qp}-\lambda b_{qp})x_{p} = - \sum _{k\in N, \atop k\neq q,p} (a_{qk}-\lambda b_{qk})x_{k}. $$

Solving for $x_{p}$ and $x_{q}$ in (2) and (3), we obtain

4 $$\begin{aligned}& \bigl((a_{pp}-\lambda b_{pp}) (a_{qq}-\lambda b_{qq}) -(a_{pq}-\lambda b_{pq}) (a_{qp}-\lambda b_{qp}) \bigr)x_{p} \\& \quad = -(a_{qq}-\lambda b_{qq}) \sum _{k\in N, \atop k\neq p,q} (a_{pk}-\lambda b_{pk})x_{k} + (a_{pq}-\lambda b_{pq})\sum_{k\in N, \atop k\neq q,p} (a_{qk}-\lambda b_{qk})x_{k} \end{aligned}$$

and

5 $$\begin{aligned}& \bigl((a_{pp}-\lambda b_{pp}) (a_{qq}-\lambda b_{qq}) -(a_{pq}-\lambda b_{pq}) (a_{qp}-\lambda b_{qp}) \bigr)x_{q} \\& \quad = -(a_{pp}-\lambda b_{pp}) \sum _{k\in N, \atop k\neq q,p} (a_{qk}-\lambda b_{qk})x_{k} + (a_{qp}-\lambda b_{qp})\sum_{k\in N, \atop k\neq p,q} (a_{pk}-\lambda b_{pk})x_{k}. \end{aligned}$$

Taking absolute values of (4) and (5) and using the triangle inequality yield

$$\begin{aligned}& \bigl\vert (a_{pp}-\lambda b_{pp}) (a_{qq}-\lambda b_{qq}) -(a_{pq}-\lambda b_{pq}) (a_{qp}- \lambda b_{qp})\bigr\vert \vert x_{p}\vert \\& \quad \leq \vert a_{qq}-\lambda b_{qq} \vert \sum _{k\in N, \atop k\neq p,q} \vert a_{pk}-\lambda b_{pk}\vert\vert x_{k} \vert + \vert a_{pq}-\lambda b_{pq} \vert \sum_{k\in N, \atop k\neq q,p} \vert a_{qk}-\lambda b_{qk}\vert\vert x_{k} \vert \end{aligned}$$

and

$$\begin{aligned}& \bigl\vert (a_{pp}-\lambda b_{pp}) (a_{qq}-\lambda b_{qq}) -(a_{pq}-\lambda b_{pq}) (a_{qp}- \lambda b_{qp})\bigr\vert \vert x_{q}\vert \\& \quad \leq \vert a_{pp}-\lambda b_{pp} \vert \sum _{k\in N, \atop k\neq q,p} \vert a_{qk}-\lambda b_{qk}\vert\vert x_{k} \vert + \vert a_{qp}-\lambda b_{qp} \vert \sum_{k\in N, \atop k\neq p,q} \vert a_{pk}-\lambda b_{pk}\vert\vert x_{k} \vert . \end{aligned}$$

Since $x_{p}\neq0$ and $x_{q}\neq0$ are, in absolute value, the largest and second largest components of *x*, respectively, we divide through by their absolute values to obtain

$$\begin{aligned}& \bigl\vert (a_{pp}-\lambda b_{pp}) (a_{qq}- \lambda b_{qq}) -(a_{pq}-\lambda b_{pq}) (a_{qp}-\lambda b_{qp}) \bigr\vert \\& \quad \leq \vert a_{qq}-\lambda b_{qq} \vert R_{p}^{q}(A,B,\lambda) + \vert a_{pq}-\lambda b_{pq} \vert R_{p}^{q}(A,B,\lambda) \end{aligned}$$

and

$$\begin{aligned}& \bigl\vert (a_{pp}-\lambda b_{pp}) (a_{qq}- \lambda b_{qq}) -(a_{pq}-\lambda b_{pq}) (a_{qp}-\lambda b_{qp}) \bigr\vert \\& \quad \leq \vert a_{pp}-\lambda b_{pp} \vert R_{q}^{p}(A,B,\lambda) + \vert a_{qp}-\lambda b_{qp} \vert R_{p}^{q}(A,B,\lambda). \end{aligned}$$

Hence,

$$\lambda\in \Bigl(\Phi_{pq}(A,B)\cap\Phi_{qp}(A,B) \Bigr) \subseteq \Phi(A,B). $$

(ii) If $x_{q}= 0$, then $x_{p}$ is the only nonzero entry of *x*. From equality (1), we have

$$A\left ( \begin{matrix} 0 \\ \vdots \\ 0 \\ x_{p}\\ 0\\ \vdots\\ 0 \end{matrix} \right )=\left ( \begin{matrix} a_{1p}x_{p} \\ \vdots \\ a_{p-1,p}x_{p} \\ a_{pp}x_{p} \\ a_{p+1,p}x_{p} \\ \vdots\\ a_{np}x_{p} \end{matrix} \right )=\lambda \left ( \begin{matrix} b_{1p}x_{p} \\ \vdots \\ b_{p-1,p}x_{p} \\ b_{pp}x_{p} \\ b_{p+1,p}x_{p} \\ \vdots\\ b_{np}x_{p} \end{matrix} \right ), $$

which implies that, for any $i\in N$, $a_{ip}=\lambda b_{ip}$, *i.e.*, $a_{ip}-\lambda b_{ip}=0$. Hence for any $i\in N$, $i\neq p$,

$$\lambda\in \Bigl(\Phi_{pi}(A,B)\cap\Phi_{ip}(A,B) \Bigr) \subseteq \Phi(A,B). $$

From (i) and (ii), $\sigma(A,B)\subseteq\Phi(A,B)$. The proof is completed. □

Since the matrix pairs $(A,B)$ and $(A^{T},B^{T})$ have the same generalized eigenvalues, we can obtain a theorem by applying Theorem 3 to $(A^{T},B^{T})$.

#### Theorem 4

*Let* $A=(a_{ij})\in\mathbb{C}^{n\times n}$, $B=(b_{ij})\in \mathbb{C}^{n\times n}$, *with* $n\geq2$, *and* $(A^{T},B^{T})$ *be a regular matrix pair*. *Then*

$$\sigma(A,B)\subseteq\Phi\bigl(A^{T},B^{T}\bigr). $$

#### Remark 1

If *B* is an identity matrix, then Theorems 3 and 4 reduce to the corresponding results of [9].

#### Remark 2

When all entries of the *i*th and *j*th rows of the matrix *B* are zero, then

$$\Phi_{ij}(A,B)= \bigl\{ z\in \mathbb{C}: \vert a_{ii}a_{jj}-a_{ij}a_{ji} \vert \leq \vert a_{jj} \vert r_{i}^{j}(A)+ \vert a_{ij} \vert r_{j}^{i}(A) \bigr\} $$

and

$$\Phi_{ji}(A,B)= \bigl\{ z\in \mathbb{C}: \vert a_{ii}a_{jj}-a_{ij}a_{ji} \vert \leq \vert a_{ii} \vert r_{j}^{i}(A)+ \vert a_{ji} \vert r_{i}^{j}(A) \bigr\} . $$

Hence, if

6 $$ \vert a_{ii}a_{jj}-a_{ij}a_{ji} \vert \leq \vert a_{jj} \vert r_{i}^{j}(A)+ \vert a_{ij} \vert r_{j}^{i}(A) $$

and

7 $$ \vert a_{ii}a_{jj}-a_{ij}a_{ji} \vert \leq \vert a_{ii} \vert r_{j}^{i}(A)+ \vert a_{ji} \vert r_{i}^{j}(A), $$

then

$$\Phi_{ij}(A,B)\cap\Phi_{ji}(A,B)=\mathbb{C}, $$

otherwise,

$$\Phi_{ij}(A,B)\cap\Phi_{ji}(A,B)=\emptyset. $$

Moreover, when inequalities (6) and (7) hold, the matrix *B* is singular, and $\operatorname{det}(A-zB)$ has degree less than *n*. As we are considering regular matrix pairs, the degree of the polynomial $\operatorname{det}(A-zB)$ has to be at least one; thus, at least one of the sets $\Phi_{ij}(A,B)\cap\Phi_{ji}(A,B)$ has to be nonempty, implying that the set $\Phi(A,B)$ of a regular matrix pair is always nonempty.

We now establish the following properties of the set $\Phi(A,B)$.

#### Theorem 5

*Let* $A=(a_{ij})$, $B=(b_{ij})\in\mathbb{C}^{n\times n}$, *with* $n\geq2$ *and* $(A,B)$ *be a regular matrix pair*. *Then the set* $\Phi_{ij}(A,B)\cap\Phi_{ji}(A,B)$ *contains zero if and only if inequalities* (6) *and* (7) *hold*.

#### Proof

The conclusion follows directly from putting $z=0$ in the inequalities of $\Phi_{ij}(A,B)$ and $\Phi_{ji}(A,B)$. □

#### Theorem 6

*Let* $A=(a_{ij})$, $B=(b_{ij})\in\mathbb{C}^{n\times n}$, *with* $n\geq2$ *and* $(A,B)$ *be a regular matrix pair*. *If there exist* $i,j \in N$, $i\neq j$, *such that*

$$\begin{gathered} b_{ii}= b_{jj}=b_{ij}=b_{ji}=0, \\ \vert a_{ii}a_{jj}-a_{ij}a_{ji} \vert \leq \vert a_{jj} \vert \sum_{k\in\beta(i), \atop k\neq i,j} \vert a_{ik} \vert + \vert a_{ij} \vert \sum _{k\in\beta(j), \atop k\neq j,i} \vert a_{jk} \vert , \end{gathered} $$

*and*

$$\vert a_{ii}a_{jj}-a_{ij}a_{ji} \vert \leq \vert a_{ii} \vert \sum_{k\in\beta(j), \atop k\neq j,i} \vert a_{jk} \vert + \vert a_{ji} \vert \sum _{k\in\beta(i), \atop k\neq i,j} \vert a_{ik} \vert , $$

*where* $\beta(i)=\{k\in N: b_{ik}=0\}$, *then* $\Phi_{ij}(A,B)\cap\Phi_{ji}(A,B)$, *and consequently* $\Phi(A, B)$ *is the entire complex plane*.

#### Proof

The conclusion follows directly from the definitions of $\Phi _{ij}(A,B)$ and $\Phi_{ji}(A,B)$. □

### Comparison with the generalized Geršgorin set

We now compare the set in Theorem 3 with the generalized Geršgorin set in Theorem 1. First, we observe two examples in which the generalized Geršgorin set is an unbounded set or the entire complex plane.

#### Example 1

Let

$$A=(a_{ij})=\left ( \begin{matrix} -1 &1 &0 &0.2 \\ 0 &1 &0.4 &0 \\ 0 &0 &i &1 \\ 0.2 &0 &0 &-i \end{matrix} \right ),\qquad B=(b_{ij})=\left ( \begin{matrix} 0.3 &0.1 &0.1 &0.1 \\ 0 &-1 &0.1 &0.1 \\ 0 &0 &i &0.1 \\ 0.1 &0 &0 &-0.2i \end{matrix} \right ). $$

It is easy to see that $b_{12}=0.1>0$ and

$$\vert b_{11} \vert =\sum_{k=2,3,4} \vert b_{1k} \vert =0.3. $$

**Figure 1:**
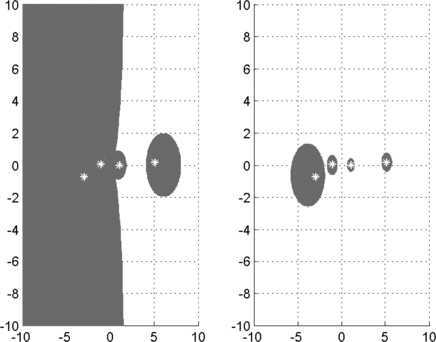
$\pmb{\Gamma(A,B)}$ **of Example** **1** **on the left, and** $\pmb{\Phi(A,B)}$ **on the right.**

Hence, from the part (i) of Theorem 2, we see that $\Gamma (A,B)$ is unbounded. However, the set $\Phi(A,B)$ in Theorem 3 is compact. These sets are given by Figure 1, where the actual generalized eigenvalues are plotted with asterisks. Clearly, $\Phi(A,B)\subset\Gamma(A,B)$.

#### Example 2

Let

$$A=(a_{ij})=\left ( \begin{matrix} -1 &1 &0 &0.2 \\ 0 &1 &0.4 &0 \\ 0 &0 &i &1 \\ 0.2 &0 &0 &-i \end{matrix} \right ),\qquad B=(b_{ij})=\left ( \begin{matrix} 0 &0 &0.1 &0.1 \\ 0 &-1 &0.1 &0.1 \\ 0 &0 &i &0.1 \\ 0.1 &0 &0 &-0.2i \end{matrix} \right ). $$

It is easy to see that $b_{11}=0$, $\beta(1)=\{2\}$ and

$$\vert a_{11} \vert =\sum_{k\in\beta(1), \atop k\neq 1} \vert a_{1k} \vert = \vert a_{12} \vert =1. $$

**Figure 2:**
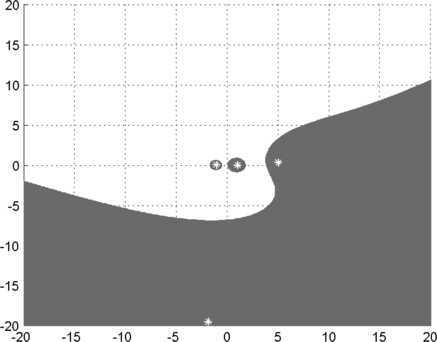
$\pmb{\Phi(A,B)}$ **of Example** **2** **.**

Hence, from the part (iii) of Theorem 2, we see that $\Gamma (A,B)$ is the entire complex plane, but the set $\Phi(A,B)$ in Theorem 3 is not. $\Phi(A,B)$ is given by Figure 2, where the actual generalized eigenvalues are plotted with asterisks.

We establish their comparison in the following.

#### Theorem 7

*Let* $A=(a_{ij})\in\mathbb{C}^{n\times n}$, $B=(b_{ij})\in\mathbb{C}^{n\times n}$, *with* $n\geq2$ *and* $(A,B)$ *be a regular matrix pair*. *Then*

$$\Phi(A,B)\subseteq\Gamma(A,B). $$

#### Proof

Let $z\in\Phi(A,B)$. Then there are $i,j \in N$, $i \neq j$ such that

$$z\in \Bigl(\Phi_{ij}(A,B) \cap\Phi_{ji}(A,B) \Bigr). $$

Next, we prove that

8 $$ \Phi_{ij}(A,B)\subseteq \Bigl(\Gamma_{i}(A,B) \cup \Gamma_{j}(A,B) \Bigr) $$

and

9 $$ \Phi_{ji}(A,B)\subseteq \Bigl(\Gamma _{i}(A,B) \cup \Gamma_{j}(A,B) \Bigr). $$

(i) For $z\in\Phi_{ij}(A,B)$, then $z\in\Gamma_{i}(A,B)$ or $z\notin \Gamma_{i}(A,B)$. If $z\in\Gamma_{i}(A,B)$, then (8) holds. If $z\notin\Gamma_{i}(A,B)$, that is,

10 $$ \vert a_{ii}-zb_{ii} \vert >R_{i}(A,B,z), $$

then

11 $$\begin{aligned}& \vert a_{jj}-z b_{jj} \vert R_{i}^{j}(A,B,z) + \vert a_{ij}-z b_{ij} \vert R_{i}^{j}(A,B,z) \\& \quad \geq \bigl\vert (a_{ii}-z b_{ii}) (a_{jj}-z b_{jj}) -(a_{ij}-zb_{ij}) (a_{ji}-z b_{ji}) \bigr\vert \\& \quad \geq \vert a_{ii}-z b_{ii} \vert\vert a_{jj} -z b_{jj} \vert - \vert a_{ij}-zb_{ij} \vert\vert a_{ji}-z b_{ji} \vert . \end{aligned}$$

Note that $R_{i}^{j}(A,B,z)=R_{i}(A,B,z)- \vert a_{ij}-z b_{ij} \vert $ and $R_{j}^{i}(A,B,z)=R_{j}(A,B,z)- \vert a_{ji}-z b_{ji} \vert $. Then from inequalities (10) and (11), we have

$$\begin{aligned}& \vert a_{jj}-z b_{jj} \vert \bigl(R_{i}(A,B,z)- \vert a_{ij}-z b_{ij} \vert \bigr) + \vert a_{ij}-z b_{ij} \vert \bigl(R_{j}(A,B,z)- \vert a_{ji}-z b_{ji} \vert \bigr) \\& \quad \geq \vert a_{jj}-z b_{jj} \vert R_{i}(A,B,z) -\vert a_{ij}-zb_{ij}\vert\vert a_{ji}-z b_{ji}\vert, \end{aligned}$$

which implies that

12 $$ \vert a_{ij}-z b_{ij} \vert R_{j}(A,B,z)\geq \vert a_{ij}-zb_{ij}\vert\vert a_{jj}-z b_{jj} \vert . $$

If $a_{ij}=z b_{ij}$, then from $z\in\Phi_{ij}(A,B)$, we have

$$\vert a_{ii}-z b_{ii}\vert\vert a_{jj}-z b_{jj} \vert \leq \vert a_{jj}-z b_{jj} \vert R_{i}^{j}(A,B,z)\leq \vert a_{jj}-z b_{jj} \vert R_{i}(A,B,z). $$

Moreover, from inequality (10), we obtain $\vert a_{jj}-z b_{jj} \vert =0$. It is obvious that

$$z\in\Gamma_{j}(A,B)\subseteq \Bigl(\Gamma_{i}(A,B)\cup \Gamma_{j}(A,B) \Bigr). $$

If $a_{ij}\neq z b_{ij}$, then from inequality (12), we have

$$\vert a_{jj}-z b_{jj} \vert \leq R_{j}(A,B,z), $$

that is,

$$z\in\Gamma_{j}(A,B)\subseteq \Bigl(\Gamma_{i}(A,B)\cup \Gamma_{j}(A,B) \Bigr). $$

Hence, (8) holds.

(ii) Similar to the proof of (i), we also see that, for $z\in \Phi_{ji}(A,B)$, (9) holds.

The conclusion follows from (i) and (ii). □

Since the matrix pairs $(A,B)$ and $(A^{T},B^{T})$ have the same generalized eigenvalues, we can obtain a theorem by applying Theorem 7 to $(A^{T},B^{T})$.

#### Theorem 8

*Let* $A=(a_{ij})\in\mathbb{C}^{n\times n}$, $B=(b_{ij})\in\mathbb{C}^{n\times n}$, *with* $n\geq2$ *and* $(A^{T},B^{T})$ *be a regular matrix pair*. *Then*

$$\Phi\bigl(A^{T},B^{T}\bigr)\subseteq\Gamma \bigl(A^{T},B^{T}\bigr). $$

#### Example 3

[1], Example 1

Let

$$A=(a_{ij})=\left ( \begin{matrix} 1 &1 &0 &0.2 \\ 0 &-1 &0.4 &0 \\ 0 &0 &i &1 \\ 0.2 &0 &0 &-i \end{matrix} \right ),\qquad B=(b_{ij})=\left ( \begin{matrix} 0.5 &0.1 &0.1 &0.1 \\ 0 &-1 &0.1 &0.1 \\ 0 &0 &i &0.1 \\ 0.1 &0 &0 &-0.5i \end{matrix} \right ). $$

**Figure 3:**
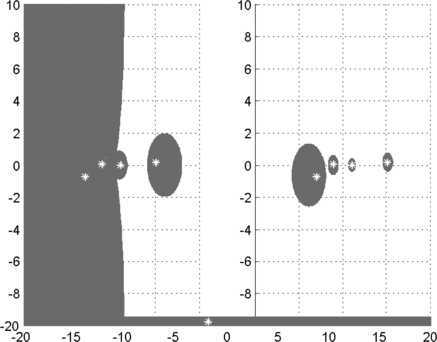
$\pmb{\Gamma(A,B)}$ **of Example** **3** **on the left, and** $\pmb{\Phi(A,B)}$ **on the right.**

It is easy to see that *B* is *SDD*. Hence, from the part (ii) of Theorem 2, we see that $\Gamma(A,B)$ is compact. $\Gamma(A,B)$ and $\Phi(A,B)$ are given by Figure 3, where the exact generalized eigenvalues are plotted with asterisks. Clearly, $\Phi(A,B)\subset\Gamma(A,B)$.

#### Remark 3

From Examples 1, 2 and 3, we see that the set in Theorem 3 is tighter than that in Theorem 1 (Theorem 7 of [1]). In addition, note that *A* and *B* in Example 1 satisfy

$$\vert a_{11} \vert =1< \sum_{k=2,3,4} \vert a_{1k} \vert =1.2 $$

and

$$\vert b_{11} \vert =\sum_{k=2,3,4} \vert b_{1k} \vert =0.3, $$

respectively. Hence, we cannot use the method in [2] to estimate the generalized eigenvalues of the matrix pair (A,B). However, the set we obtain is very compact.

## Conclusions

In this paper, we present a new generalized eigenvalue localization set $\Phi(A,B)$, and we establish the comparison of the sets $\Phi(A,B)$ and $\Gamma(A,B)$ in Theorem 7 of [1], that is, $\Phi(A,B)$ captures all generalized eigenvalues more precisely than $\Gamma(A,B)$, which is shown by three numerical examples.
